# Antibiotic use influences outcomes in advanced pancreatic adenocarcinoma patients

**DOI:** 10.1002/cam4.3870

**Published:** 2021-07-11

**Authors:** Chirayu Mohindroo, Merve Hasanov, Jane E. Rogers, Wenli Dong, Laura R. Prakash, Seyda Baydogan, Jonathan D. Mizrahi, Michael J. Overman, Gauri R. Varadhachary, Robert A. Wolff, Milind M. Javle, David R. Fogelman, Michael T. Lotze, Michael P. Kim, Matthew H.G. Katz, Shubham Pant, Ching‐Wei D. Tzeng, Florencia McAllister

**Affiliations:** ^1^ Department of Clinical Cancer Prevention The University of Texas MD Anderson Cancer Center Houston Texas USA; ^2^ Pharmacy Clinical Programs The University of Texas MD Anderson Cancer Center Houston Texas USA; ^3^ Department of Biostatistics The University of Texas MD Anderson Cancer Center Houston Texas USA; ^4^ Department of Surgical Oncology The University of Texas MD Anderson Cancer Center Houston Texas USA; ^5^ Department of Cancer Medicine The University of Texas MD Anderson Cancer Center Houston Texas USA; ^6^ Department of Gastrointestinal Medical Oncology The University of Texas MD Anderson Cancer Center Houston Texas USA; ^7^ Department of Surgery University of Pittsburgh Medical Center Pittsburgh Pennsylvania USA; ^8^ Department of Investigation Cancer Therapeutics The University of Texas MD Anderson Cancer Center Houston Texas USA

**Keywords:** antibiotics, autophagy, chemotherapeutic agents, immunity, microbiota, pancreatic adenocarcinoma

## Abstract

Recent studies defined a potentially important role of the microbiome in modulating pancreatic ductal adenocarcinoma (PDAC) and responses to therapies. We hypothesized that antibiotic usage may predict outcomes in patients with PDAC.

We retrospectively analyzed clinical data of patients with resectable or metastatic PDAC seen at MD Anderson Cancer from 2003 to 2017. Demographic, chemotherapy regimen and antibiotic use, duration, type, and reason for indication were recorded.

A total of 580 patients with PDAC were studied, 342 resected and 238 metastatic patients, selected retrospectively from our database. Antibiotic use, for longer than 48 hrs, was detected in 209 resected patients (61%) and 195 metastatic ones (62%). On resectable patients, we did not find differences in overall survival (OS) or progression‐free survival (PFS), based on antibiotic intake. However, in the metastatic cohort, antibiotic consumption was associated with a significantly longer OS (13.3 months vs. 9.0 months, HR 0.48, 95% CI 0.34–0.7, *p* = 0.0001) and PFS (4.4 months vs. 2 months, HR 0.48, 95% CI 0.34–0.68, *p *= <0.0001). In multivariate analysis, the impact of ATB remained significant for PFS (HR 0.59, *p* = 0.005) and borderline statistically significant for OS (HR 0.69, *p* = 0.06). When we analyzed by chemotherapy regimen, we found that patients who received gemcitabine‐based chemotherapy as first‐line therapy (n = 118) had significantly prolonged OS (HR 0.4, *p* 0.0013) and PFS (HR 0.55, *p* 0.02) if they received antibiotics, while those receiving 5FU‐based chemotherapy (n = 98) had only prolonged PFS (HR 0.54, *p* = 0.03).

Antibiotics‐associated modulation of the microbiome is associated with better outcomes in patients with metastatic PDAC.

## Abstract 2

We have analyzed the effect of antibiotics’ intake on two cohorts of patients with pancreatic adenocarcinoma, resectable, and metastatic. We have found that on the metastatic cohort, antibiotics use was significantly associated with better outcomes, particularly, on patients that received gemcitabine based‐chemotherapy as the first line.

## 1 INTRODUCTION

It is estimated that in 2020, there will be 57,600 new cases of pancreatic adenocarcinoma (PDAC), and approximately 47,050 deaths. The 5‐year survival rate of PDAC is 9.3% making it is the third most common cause of cancer‐related death in the United States.^1^ The dismal prognosis has been attributed to the lack of early detection, aggressive biology, and the absence of effective therapies. Recent studies have shown that PDAC‐associated gut and tumor microbiome can play a significant role in disease progression and responses to therapy in preclinical models.^2^, ^3^ Bacterial diminution via antibiotic administration in PDAC orthotopic mouse models altered the tumor microenvironment, resulting in higher T cell activation and ultimately improved immunosurveillance and increased sensitivity to immunotherapy.^2^, ^4^

The presence of specific microbes within tumors is linked to resistance to gemcitabine as a consequence of the metabolism of this chemotherapeutic agent.^5^ One of those microbes, gammaproteobacteria is highly prevalent in human PDAC. Recent studies from our group have further identified a specific microbial signature in tumors from PDAC long‐term survivors.^6^ We found that higher tumor alpha‐diversity correlated with better outcomes.^6^ One could expect that changes in the microbiome by the administration of antibiotics could result in differential outcomes.

Antibiotics can alter the gut microbiota diversity and composition, leading to modified responses to chemotherapeutic agents and immunotherapy regimens.^5^, ^7^, ^8^, ^9^ There have been several recent studies examining the effect of antibiotic use in patients with solid tumors on immune checkpoint inhibitor treatment. Through retrospective studies as well as meta‐analyses, some studies revealed that antibiotics intake during the period of immunotherapy initiation was associated with worse overall and progression‐free survival in several tumor types including renal cell carcinoma, non‐small cell lung cancer, melanoma, sarcoma, hepatocellular carcinoma, urothelial carcinoma, and GI stromal tumors.^8^, ^10^, ^11^, ^12^, ^13^, ^14^ In metastatic colorectal cancer patients, antibiotic use prior to starting 5FU‐based chemotherapy was associated with worse OS and PFS.^15^ In contrast, antibiotic use in metastatic colorectal cancer patients treated with bevacizumab is associated with decreased mortality.^16^

Antibiotics have been linked to lower efficacy of immunotherapy in several solid tumors, but none of these studies specifically examined PDAC.^7^, ^8^, ^9^ While most studies suggest that lower alpha‐diversity of the gut microbiome is linked to worse responses to immunotherapy, preclinical studies in PDAC have suggested a potential positive role of antibiotics in disease progression and therapy responses.^2^, ^3^, ^5^ Systematic assessment of the potential value of antibiotic administration in patients with pancreatic cancer remains to be performed. In this retrospective study, we evaluated the effect of antibiotics on outcomes in patients with resected and metastatic pancreatic cancer.

## 2 METHODS

### 2.1 Patients and antibiotic use

We studied 580 patients with evaluable chemotherapy data belonging to the following two groups: (1) Patients with localized/borderline disease that underwent resection (n = 342) and (2) Patients with metastatic disease (n = 238). Patients with resectable or metastatic disease diagnosed and/or treated at MD Anderson Cancer Center between 2003–2015 and 2009–2017, respectively, were included in the analysis.

We reviewed patients’ medical records and collected data on patient demographics, antibiotic use, duration, class and indication, disease and treatment characteristics, and survival data including progression, death, and last follow‐up dates. Antibiotic use was assessed from diagnosis to death or the date of the last follow‐up for metastatic cohort, and diagnosis to surgical resection for the resected cohort. All patients were followed up until death or data lock (February 2019 for both cohorts). Antibiotic use was defined as any use of antibiotics for longer than 48 hrs, which excludes the usual one‐time antibiotic given before or during procedure or surgery. Information regarding the class of the antibiotics and indication of use were also provided. This study was approved by the institutional review board.

### 2.2 Statistical analysis

Patients characteristics were described according to antibiotics intake and compared using Fisher or chi‐squared test for categorical data and Wilcoxon rank‐sum test for continuous data. Frequencies and percentages were reported for categorical variables. Summary statistics such as median, minimum, and maximum were provided for continuous data (such as age). Overall survival (OS) was defined as the time from diagnosis to death from any cause. Patients were censored to the date of the last follow‐up. Progression‐free survival (PFS) was defined as the time from surgery to progression or death, whichever occurred first, in the resected patients; and as the time from first‐line chemotherapy to progression or death, whichever occurred first, in the metastatic group.

Univariate and multivariable Cox proportional hazard models were used to determine the effects of potential risk factors and antibiotic status variables on OS and PFS. Clinically and statistically important variables were included in the multivariable models. Hazard ratios and 95% confidence intervals were provided. Kaplan–Meier curves were estimated for the survival distributions by antibiotic status. The Log‐rank test was used to test the difference in survival distributions between treatment groups. All tests were two‐sided. P‐values less than 0.05 are considered statistically significant. All analyses were conducted using SAS 9.4 (SAS, Cary, NC) and S‐Plus 8.0 (TIBCO Software Inc., Palo Alto, CA) software.

## 3 RESULTS

### 3.1 Demographical and clinical characteristics

A total of 580 patients were studied: 342 patients with resected PDAC and 238 patients with metastatic PDAC. All patients were seen between 2003 and 2017 at a single institution: University of Texas MD Anderson Cancer Center.

#### 3.1.1 Resected Cohort

The median age was 64 years (range of 34–85 years), including 147 (43%) females and 195 (57%) males. A total of 284 (83%) patients in the cohort were White followed by 33 (10%) Hispanic, 10 (3%) Black, 8 (2%) Asian, and 7 (2%) from other racial backgrounds. Most patients had PDAC Stage IIB (n = 185, 54%), followed by IIA (n = 111, 32%), Stage 0/I (n = 37,11%), and 9 (3%) patients were found to be stage III/IV during surgical exploration (Table 1).

**TABLE 1:** Baseline characteristics of both resectable and metastatic pancreatic ductal adenocarcinoma cohorts

Resectable Cohort				
	Total (n = 342)	Antibiotics (n = 209)	No antibiotics (n = 133)	*p* Value
**Age at diagnosis‐median (range)**	64 (34–85)	65 (38 – 85)	62 (34 – 83)	0.0085
**Gender, n (%)**				
Female	147 (43%)	88 (42%)	59 (44%)	0.6812
Male	195 (57%)	121 (58%)	74 (56%)	
**Race, n (%)**				
White	284 (83%)	177 (85%)	107 (80%)	0.7992
Black	10 (3%)	6 (3%)	4 (3%)	
Hispanic	33 (10%)	17 (8%)	16 (13%)	
Asian	8 (2%)	5 (2%)	3(2%)	
Others	7 (2%)	4 (2%)	3(2%)	
**Stage, n (%)**				
0/I	37 (11%)	17 (8%)	20 (15%)	0.2519
IIA	111 (32%)	69 (33%)	42 (32%)	
IIB	185 (54%)	117 (56%)	68 (51%)	
III/IV	9 (3%)	6 (3%)	3 (2%)	
**Type of Chemotherapy, n (%)**				
5FU Based	44 (13%)	34 (16%)	10 (8%)	0.0533
Gemcitabine	206 (60%)	118 (56%)	88 (66%)	
Gemcitabine and 5FU	7 (2%)	3 (2%)	4 (3%)	
No chemotherapy	85 (25%)	54 (26%)	31 (23%)	
**Metastatic Cohort**				
	**Total (n = 238)**	**Antibiotics (n = 195)**	**No antibiotics (n = 43)**	***p* Value**
**Age at Diagnosis‐Median (range)**	64 (25–84)	62 (25–84)	65 (46–83)	0.31
**Gender, n (%)**				
Female	117 (49%)	98 (50%)	19 (44%)	0.47
Male	121 (51%)	97 (50%)	24 (56%)	
**Race, n (%)**				
White	176 (74%)	140 (72%)	36 (85%)	0.73
Black	22 (9%)	19 (10%)	3 (7%)	
Hispanic	10 (4%)	9 (5%)	1 (2%)	
Asian	11 (5%)	10 (5%)	1 (2%)	
Others	8 (3%)	7 (3%)	1 (2%)	
Not known	11 (5%)	10 (5%)	1 (2%)	
**Site of the Disease, n (%)**				
Head	111 (47%)	98 (50%)	13 (30%)	0.058
Body & Neck	76 (32%)	58 (30%)	18 (42%)	
Tail	51 (21%)	39 (20%)	12 (28%)	
**Type of Chemotherapy, n (%)**				
5FU Based	98 (41%)	78 (40%)	20 (47%)	0.68
Gemcitabine	118 (50%)	97 (50%)	21 (49%)	
Other	22 (9%)	20 (10%)	2 (4%)	

#### 3.1.2 Metastatic Cohort

Median age was also 64 years (range of 25–84 years) for the patients in this group, including 117 (49%) females and 121 (51%) males. A total of 176 (74%) patients in the cohort were White, 22 (9%) Black, 11 (5%) Asian, 10 (4%) Hispanic, and 8 (3%) from other races. The most common location for the primary tumor was the head of pancreas in 111(47%) patients, followed by body and neck in 76 (32%) patients, with 51 (21%) patients having the primary tumor in the tail (Table 1).

### 3.2 Antibiotics intake description

#### 3.2.1 Resected Cohort

The frequency of antibiotic use from diagnosis to surgical resection was 61% (n = 209). Single dose of antibiotics administered prior to operation or ancillary procedures was not considered for this analysis. While 133 patients (39%) did not receive any antibiotics, 209 patients (61%) received antibiotics prior to surgery. Within the group received antibiotics (209 patients, 61% of total resected cohort), 62 patients (18%) received less than 7 days, 147 patients (43%) received 7 days or more duration of antibiotics. A total of 206 (60%) patients received gemcitabine‐based chemotherapy (mostly gemcitabine/Nab‐paclitaxel), while 44 (13%) received 5‐fluorouracil‐based chemotherapy, and 7 (2%) both chemotherapies. Eighty‐five patients (25%) did not receive chemotherapy prior to surgical exploration and resection (Table 1). The following antibiotics were received by the resected PDAC patients: quinolones (n = 168), beta‐lactams (n = 80), nitroimidazoles (n = 48), glycopeptides (n = 32), tetracyclines (n = 18), macrolides (n = 14), and sulfa drugs (n = 4) (Table S1).

#### 3.2.2 Metastatic Cohort

The frequency of antibiotic use from diagnosis to death or the date of the last follow‐up was 82% (n = 195). Forty‐three patients (18%) did not receive any antibiotics. Among 195 patients (82%) that received antibiotics, 35 patients (15%) received antibiotics less than 7 days, and 160 patients (67%) received antibiotics 7 days and more. A total of 118 (50%) patients received gemcitabine‐based chemotherapy, while 98 (41%) received 5‐Fluorouracil‐based chemotherapy, and 22 (9%) received other chemotherapeutic regimens in the first‐line setting (Table 1). The antibiotics received by metastatic PDAC patients were as follows: quinolones (n = 141), beta‐lactams (n = 135), glycopeptides (n = 59), nitroimidazoles (n = 51), macrolides (n = 31), tetracyclines (n = 27), and sulfa drugs (n = 21). (Tabls S1).

### 3.3 Influence of antibiotics use in clinical outcomes

#### 3.3.1 Resected Cohort

**FIGURE 1:**
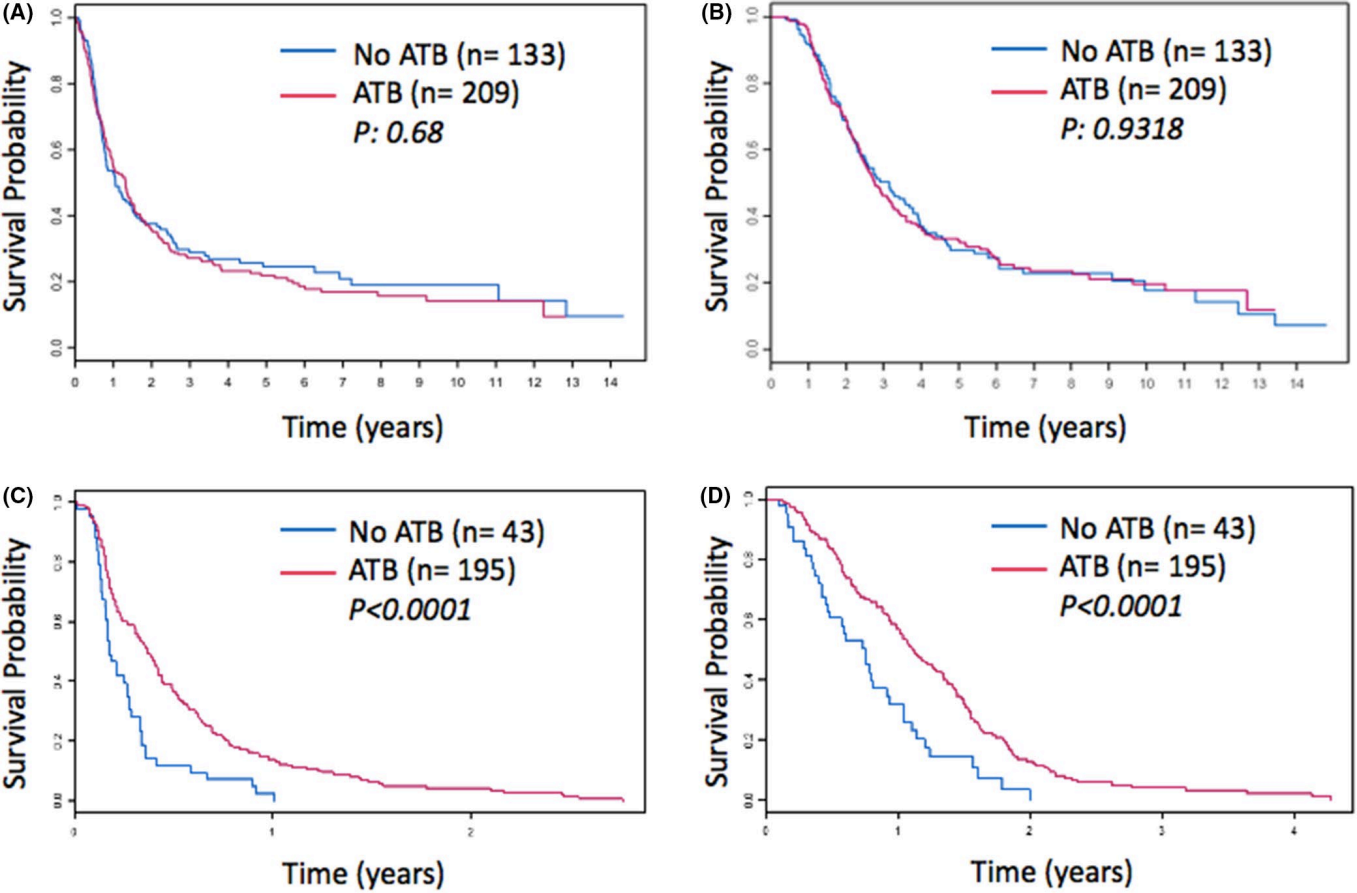
Kaplan–Meier Survival Curves of resectable and metastatic PDAC cohorts according to antibiotic use. PFS (A) and OS (B) in resectable cohort. PFS (C) and OS (D) in metastatic cohort

The median PFS of patients who took antibiotics prior to surgery was 12.3 months versus 10.2 months in those who did not (HR 0.95, 95% CI 0.74–1.22, *p* = 0.68; Table 2, Figure 1A), while OS was 32.7 months versus 32.8 months (HR 0.99, 95% CI 0.76–1.28, *p* = 0.93; Table 2, Figure 1B) on univariate analysis. Therefore, antibiotics intake prior to surgery did not affect outcomes in those patients who underwent surgical resection.

**TABLE 2:** Univariate analysis for OS and PFS in resectable and metastatic cohorts

Resectable Cohort (n = 342)					
		Overall survival (OS)		Progressive‐free survival (PFS)	
Prognostic factors	n (%)	OS HR (95% CI)	*p* value	PFS HR (95% CI)	*p* value
**Gender**					
Female	147 (43)	0.82 (0.64–1.06)	0.13	0.83 (0.65–1.07)	0.15
Male	195 (57)				
**Age**					
</=65	193 (56)	0.94(0.73–1.21)	0.64	1.04 (0.82–1.33)	0.73
>65	149 (44)				
**Antibiotics received**	209 (61)	0.99 (0.76–1.28)	0.93	0.95 (0.74–1.22)	0.68
**Intra‐abdominal infections**	133 (39)	1.13 (0.88–1.47)	0.34	1.06 (0.83–1.35)	0.65
**Urinary Infections**	30 (9)	1.04 (0.67–1.61)	0.88	1.03 (0.67–1.56)	0.91
**Respiratory Infections**	26 (8)	0.83 (0.53–1.32)	0.43	0.75 (0.48–1.17)	0.21
**Skin/Soft Tissue Infections**	30 (9)	0.78 (0.51–1.20)	0.26	0.87 (0.58–1.31)	0.51
**Blood Infections**	8 (2)	1.17 (0.48–2.83)	0.73	1.15 (0.48–2.79)	0.75
**Other Infections**	62 (18)	0.91 (0.66–1.26)	0.59	0.86 (0.64–1.17)	0.34
**Metastatic Cohort (n = 238)**					
		**Overall Survival (OS)**		**Progressive‐Free Survival (PFS)**	
**Prognostic factors**	**n (%)**	**OS HR (95% CI)**	**P value**	**PFS HR (95% CI)**	**P value**
**Gender**					
Female	117(49)	0.73(0.56–0.97)	**0.0327**	0.97(0.75–1.26)	0.82
Male	121(51)				
**Age**					
</=65	131(55)	1.09(0.83–1.44)	0.52	1.09(0.84–1.41)	0.49
>65	107(45)				
**Antibiotics Received**	195(82)	0.49(0.34– 0.7)	**0.0001**	0.48(0.34–0.68)	**<0.0001**
**Intra‐abdominal Infections**	102(43)	0.79(0.6 – 1)	0.1	0.86(0.66–1.12)	0.28
**Urinary Infections**	55(23)	0.83(0.61–1.15)	0.27	0.83(0.61–1.13)	0.25
**Respiratory Infections**	81(34)	0.58(0.43–0.78)	**0.0003**	0.64(0.48–0.84)	**0.0017**
**Skin/Soft Tissue Infections**	39(16)	0.55(0.37–0.81)	**0.0028**	0.60(0.42–0.85)	**0.0047**
**Blood Infections**	37(16)	1.13(0.78–1.64)	0.5	0.89(0.62–1.27)	0.52
**Other Infections**	92(39)	0.75(0.56–1.00)	0.052	0.8(0.61–1.05)	0.11

#### 3.3.2 Metastatic Cohort

The use of ATB was associated with significantly longer PFS (4.4 months vs. 2 months, HR 0.48, 95% CI 0.34–0.68, *p* = <0.0001; Table 3, Figure 1C) and median OS (13.3 months vs. 9.0 months, HR 0.48, 95% CI 0.34– 0.7, *p* = 0.0001; Table 3, Figure 1D) in metastatic patients.

**TABLE 3:** Multivariate analysis for OS and PFS in the metastatic cohort

Prognostic factors	OS HR (95% CI)	*p* value	PFS HR (95% CI)	*p* value
Antibiotics received	0.69(0.47–1.02)	**0.06**	0.59(0.41–0.85)	**0.005**
Age	0.94 (0.98–1)	0.41	0.99(0.97– 1)	0.18
Gender	0.75(0.57–1)	**0.05**	0.96(0.74–1.24)	0.76
Respiratory infections	0.63(0.45–0.86)	**0.0045**	0.74(0.5–1)	0.052
Skin Infections	0.56(0.38–0.84)	**0.0057**	0.65(0.45–0.93)	**0.02**

Given these results, we carried out multivariate analysis to assess the robustness of this finding accounting for other significant variables on the univariate analysis (gender, age, respiratory infections, and skin/soft tissue infections). Antibiotics use remained independently associated with significantly prolonged PFS (HR 0.59, *p* = 0.005), while for OS antibiotics usage was associated with borderline statistically significant OS (HR 0.69, *p* = 0.06). On univariate analysis respiratory and skin infections were associated with an improved outcome. Furthermore, on multivariate analysis respiratory and skin infections independently remained associated with an improved OS and PFS (Table 3).

### 3.4 Influence of antibiotics by chemotherapy type

We next examined if antibiotics differentially affected survival based on the type of chemotherapy that patients received as first‐line within the metastatic cohort.

**FIGURE 2:**
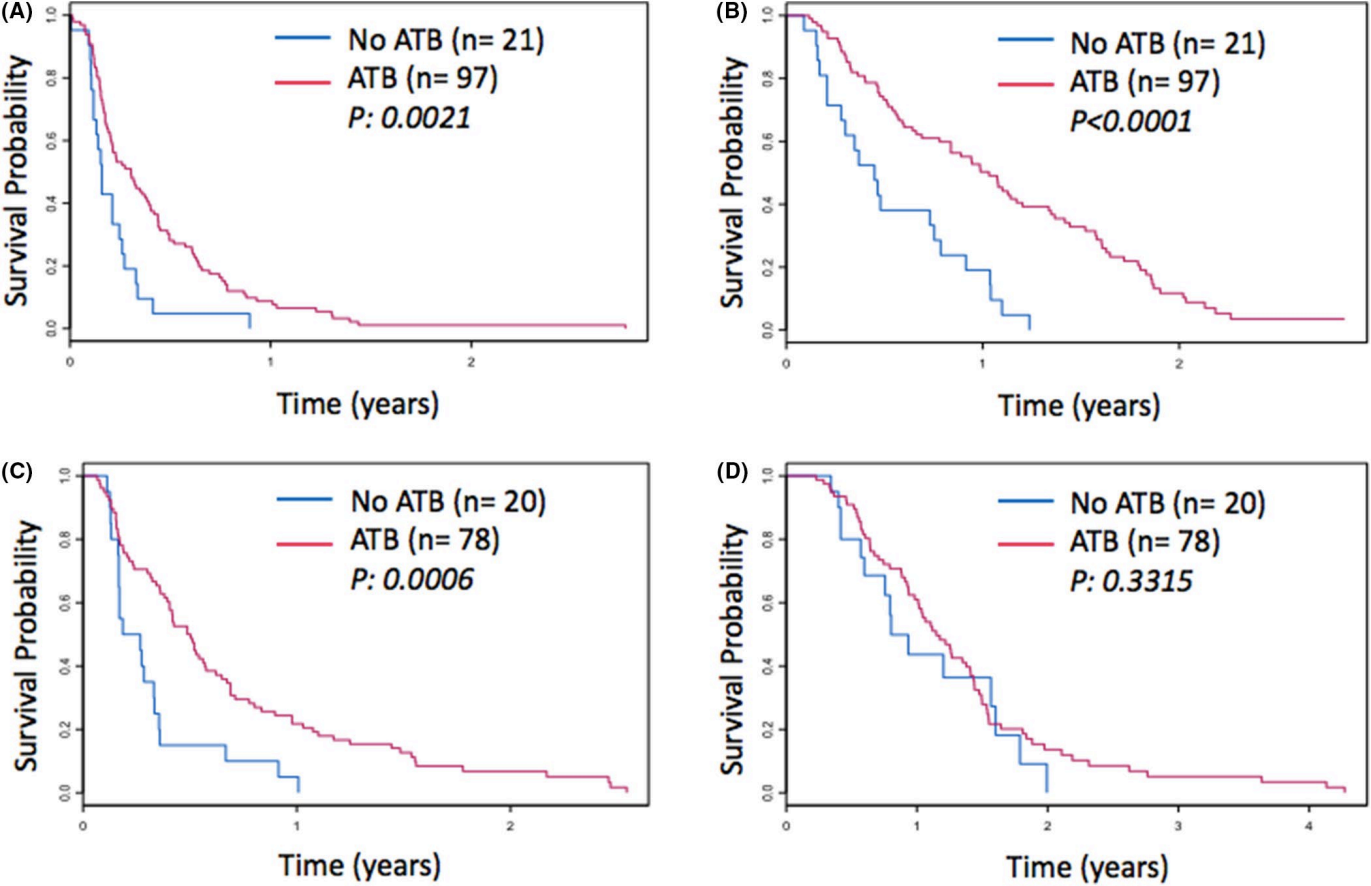
Kaplan–Meier Survival Curves of subgroup analysis per chemotherapy type in metastatic PDAC cohort according to antibiotic use. PFS (A) and OS (B) for Gemcitabine‐ based chemotherapy group. PFS (C) and OS (D) for 5FU‐based chemotherapy group

To this end, we further analyzed outcomes in patients receiving Gemcitabine‐based chemotherapy (n = 118) and 5‐FU based chemotherapy (n = 98). After applying the multivariate modeling in the gemcitabine‐based subgroup, patients who received antibiotics had a significantly improved PFS (HR 0.55, *P* 0.02) and OS (HR 0.4, P 0.0013); (Table 4, Figure 2A, Figure 2B). For the FOLFIRINOX group, following multivariate analysis, patients who had received ATB also had a significantly prolonged PFS (HR 0.54, *p* = 0.003), but did not have a significantly different OS (HR 1.17, *p* = 0.59) (Table 4, Figure 2C, D).

**TABLE 4:** Subgroup analysis in the metastatic PDAC cohort based on chemotherapy type

Gemcitabine based (n = 118)				
Prognostic factors	OS HR (95% CI)	***p*** value	PFS HR (95% CI)	***p*** value
Antibiotics received	0.4(0.23–0.7)	**0.0013**	0.55(0.33–0.94)	**0.02**
Age	0.98(0.96–1)	0.14	0.99(0.97–1.01)	0.48
Gender	0.92(0.61–1.39)	0.69	0.91(0.62–1.33)	0.64
Respiratory infections	0.67(0.42–1.07)	0.09	0.76(0.49–1.16)	0.2
Skin infections	0.8(0.45–1.41)	0.44	0.74(0.44–1.25)	0.27
**5FU‐based (n = 98)**				
**Prognostic factors**	**OS HR (95% CI)**	***p* value**	**PFS HR (95% CI)**	***p* value**
Antibiotics received	1.17(0.64–2.14)	0.59	0.54(0.31–0.94)	**0.03**
Age	0.98(0.96–1)	0.39	0.97(0.95–0.99)	**0.03**
Gender	0.62(0.39–0.98)	0.04	0.87(0.57–1.32)	0.52
Respiratory infections	0.55(0.32–0.93)	0.02	0.69(0.42–1.19)	0.13
Skin infections	0.42(0.22–0.82)	0.01	0.44(0.24–0.81)	**0.008**

## 4 DISCUSSION

The present study reports the impact of antibiotic usage on overall survival and progression‐free survival in PDAC patients, contrasting results in early resected patients and those with metastatic disease. To the best of our knowledge, this is the first study that systemically examined the effect of ATB usage in a large population of pancreatic cancer patients with resectable and metastatic disease. Use of antibiotics in patients with metastatic PDAC was associated with better outcomes: increased PFS and OS and only PFS after multivariate analysis. Furthermore, subgroup analysis revealed that antibiotic usage in patients who received gemcitabine independently prolonged OS and PFS. In the 5FU‐based chemotherapy group, patients who had received ATB had a significantly prolonged PFS but no differences in OS.

Antibiotics are known to reduce microbial diversity and have been associated with worse outcomes in the setting of immunotherapy for other tumor types.^8^, ^10^, ^11^, ^12^, ^13^, ^14^ Based on preclinical pancreatic cancer studies ^2^, ^3^ and outcomes from this retrospective clinical study, antibiotics may be playing a potentially beneficial role in PDAC. Gammaproteobacteria (GP) in tumors can affect gemcitabine activity, converting gemcitabine (20,20‐difluorodeoxycytidine) into its inactive form (20,20 ‐difluorodeoxyuridine), implying that the tumor microbiome in PDAC may be responsible for the tumor resistance to gemcitabine.^5^ The use of antibiotics may result in decreased proportions of those drug inactivating‐bacteria which would result in higher chemotherapy activation and improved outcomes.^5^ Better responses to gemcitabine (27.6 vs. 15.1%), and clinically better OS (13.83 vs. 7.53 months), and PFS (4.9 vs. 2.5 months) were observed in patients that received antibiotics with several solid tumors that were examined including PDAC.^17^ Besides altering the microbiome, there are multiple other mechanisms antibiotics could prolong the survival in PDAC patients at a cellular level. Antibiotics alone or in combination with other chemotherapeutic agents could act as anti‐neoplastic agents, by cytotoxic mechanisms such as loss of STAT 3 activation ^18^ or inducing direct morphologic changes such as a decrease of microvilli‐like protrusions on cell surface membrane and the swelling of mitochondria.^19^ Antibiotics could also be involved in reducing the expression of proteins such as CD 47, which would promote macrophage and tumor cell interaction, ultimately leading to tumor cell death.^19^

Immune response modulation by infections, especially chronic infections, and tumors has similarities ^20^ causing an immune‐suppressive microenvironment. Evasion of the infection with antibiotics might undo the immunosuppressive microenvironment that would otherwise promote tumor growth.

Use of antibiotics in resectable patients was not associated with better outcome, which may be explained by the fact that surgery‐related outcomes may play a more important role with other prognostic factors in resected patients (surgical margins, pathological response, lymph node invasion, nerve or vascular invasion).^21^, ^22^ A biologic understanding of which patients require antibiotics and those who do not in the metastatic setting is not immediately forthcoming. It is known that patients with a low neutrophil/lymphocyte ratio predict for better outcome in patients on Nab‐paclitaxel regimens.^23^, ^24^, ^25^ Furthermore, our studies with neoadjuvant administration of gemcitabine‐containing regimens along with autophagy inhibition with hydroxychloroquine administration revealed improved outcomes.^26^, ^27^, ^28^ It would be interesting to retrospectively examine completed randomized studies to determine whether antibiotic usage defined better outcomes.^29^, ^30^ An increased neutrophil to lymphocyte ratio may predict which patients might best respond to a course of antibiotics prior to chemotherapy, suggesting the possibility of necrosis and/or occult infection.^31^, ^32^, ^33^, ^34^, ^35^, ^36^, ^37^, ^38^, ^39^, ^40^, ^41^, ^42^, ^43^, ^44^, ^45^, ^46^, ^47^, ^48^, ^49^, ^50^

Our study has several limitations. First, the study was a single‐center study of retrospective nature. Second, the analysis did not take into consideration additional factors which could alter the microbiome composition such as diet, country of origin, other medications or supplements. We conducted a multivariate and subgroup analysis to avoid confounding factors within our data set that replicated similar results after adjustment with age, gender, initial treatment type and the reason for antibiotic use. It would be worth mentioning here, there has been increasing evidence that probiotics could possibly prolong survival in PDAC by altering the microbiome.^51^ However, due to the retrospective nature of our study and the fact that probiotics are often not documented in the medication list during both outpatient visits and inpatient admissions, we were not able to capture this data for our study. In the resected PDAC cohort, we collected the antibiotic exposure data prior to surgical extirpation, not after. It is unclear if post‐surgical antibiotic use affects the survival of the patients, and there is no available correlative data in the literature examining this measure.

Finally, recent studies have described differences in the microbiome of individuals of different races and ethnicity.^52^ Therefore, a clear study limitation is the enrichment of patients of the white race in both resected and metastatic PDAC groups. While the data strongly support the benefit of antibiotics consumption in PDAC patients, there could be alternative explanations for this association. Antibiotics may improve prognosis by limiting local and systemic infections, completely independent of their effect on the tumor and its microenvironment. Also, there may be survivor bias since patients living longer may also have more opportunities to receive any type of therapy, besides antibiotics.

In conclusion, antibiotic use among patients with metastatic PDAC is associated with an increased progression‐free and overall survival, even more marked on patients who received concomitant gemcitabine‐based chemotherapy. These findings need to be validated within a larger group of patients within the context of a prospective randomized clinical trial that is being planned now (MTL).

## CONFLICT OF INERTEST

No conflict of interests reported by authors.

## AUTHORS’ CONTRIBUTIONS

CM, MH, and FM designed, extracted data, performed analysis, and wrote the manuscript. FM supervised the study. WD performed statistical analysis. JR, LP, SB, and JM had worked on data tabulation and extraction. MO, GV, RW, MJ, DF, MK, MK, SP, CWT, and FM contributed with patients’ data, study revision, analysis, and writing revision. ML contributed by the critical analysis of manuscript and contribution in resubmission write up.

## Supporting information

Table S1
