# Supplementary material for: Antibiotic use influences outcomes in advanced pancreatic adenocarcinoma patients
Source: Cancer Med. 2021 Jul 11;10(15):5041–50. doi: 10.1002/cam4.3870 (PMC8335807; doi:10.1002/cam4.3870)
Supplement: Supplementary file 1 — Table S1 [file CAM4-10-5041-s001.docx]

**Supplementary Table 1**

| **Distribution of Type Antibiotics** | | |
| --- | --- | --- |
| **Type of Antibiotics** | **Metastatic Cohort (n=238)** | **Resectable Cohort**  **(n=342)** |
| Beta Lactams | 135(57%) | 80(23%) |
| Quinolones | 141(59%) | 168(49%) |
| Nitroimidazole | 51(21%) | 48(14%) |
| Macrolides | 31(13%) | 14(4%) |
| Glycopeptides | 59(25%) | 32(9%) |
| Tetracylines | 27(11%) | 18(5%) |
| Sulfa Drugs | 21(9%) | 4(1%) |
| Others | 69(29%) | 39(11%) |
